# TRAIL and FasL Functions in Cancer and Autoimmune Diseases: Towards an Increasing Complexity

**DOI:** 10.3390/cancers11050639

**Published:** 2019-05-08

**Authors:** Aurélie Rossin, Giorgia Miloro, Anne-Odile Hueber

**Affiliations:** Université Côte d’Azur, CNRS, Inserm, iBV, 06108 Nice, France; gmiloro@unice.fr

**Keywords:** death receptors, autoimmunity, cancer, immune system, cell death

## Abstract

Tumor Necrosis Factor-Related Apoptosis Inducing Ligand (TRAIL/TNFSF10) and Fas Ligand (FasL/TNFSF6), two major cytokines of the TNF (Tumor Necrosis Factor) superfamily, exert their main functions from the immune system compartment. Mice model studies revealed that TRAIL and FasL-mediated signalling both control the homeostasis of the immune cells, mainly from the lymphoid lineage, and function on cytotoxic cells as effector proteins to eliminate the compromised cells. The first clues in the physiological functions of TRAIL arose from the analysis of TRAIL deficient mice, which, even though they are viable and fertile, are prone to cancer and autoimmune diseases development, revealing TRAIL as an important safeguard against autoimmunity and cancer. The naturally occurring gld (generalized lymphoproliferative disease) and lpr (lymphoproliferation) mutant mice develop lymphadenopathy and lupus-like autoimmune disease. The discovery that they are mutated in the fasl and the fas receptor gene, respectively, demonstrates the critical role of the FasL/Fas system in lymphocyte homeostasis and autoimmunity. This review summarizes the state of current knowledge regarding the key death and non-death immune functions that TRAIL and FasL play in the initiation and progression of cancer and autoimmune diseases.

## 1. Introduction

Human Tumor Necrosis Factor-Related Apoptosis Inducing Ligand (TRAIL), which was cloned in 1995 as the third death inducing ligand of the Tumor Necrosis Factor (TNF) superfamily (TNFSF), shares 28% and 23% homology with Fas Ligand (FasL) and Tumor Necrosis Factor α (TNFα), respectively [1]. TRAIL and FasL are type II membrane proteins that exert optimal biologic activity in a trimeric form [2]. Both can be further cleaved by endopeptidases (metalloproteases for FasL or cathepsin E and cysteine protease for TRAIL) producing soluble trimer versions of the ligands [3,4,5]. Membrane bound forms are more potent in death induction [6,7,8], but soluble proteins are also biologically active, and soluble FasL was reported to exert alternative non-death functions [9]. The human TRAIL receptor system is the most complex of the two: TRAIL binds to five different receptors: the two membrane receptors TRAIL-R1 (DR4/TNFRSF10A) and TRAIL-R2 (DR5/TNFRSF10B) contain a full length cytoplasmic domain that harbors an intracellular death domain (DD), whereas the receptors TRAIL-R3 (TNFRSF10C) and TRAIL-R4 (TNFRSF10D) show a truncated intracellular part and, in the absence of death domain, might function as decoy receptors [10]. Lastly, osteoprotegerin (OPG/TNFRSF11B), which was initially reported as ligand for RANK (receptor activator of nuclear factor κ) (TNFRSF11A), was described as a TRAIL soluble receptor [10]. For its part, human FasL binds to one DD-containing membrane receptor, Fas (CD95/TNFRSF6), and to one soluble decoy receptor (DcR3) [10]. On their side, all Fas and TRAIL receptors exist as ligand-independent homotrimers rather than as monomer receptors, thanks to the presence of a preligand assembly domain in their extracellular region [2]. Interestingly, ligand independent heteromers between some TNFRS members (such as TRAIL-R, Fas, and CD40) were also reported, suggesting that hetero interaction might be a way to modulate the initial signalling steps [11]. The TRAIL and the FasL signalling regulation can differ appreciably between species, which emphasizes that a particular attention should be paid before transposing data obtained in mice into human contexts, as rodents only possess one DD-containing TRAIL receptor and no FasL decoy receptor, and that the primary sequence of the human and mice protein of each receptor is quite divergent.

The expression of FasL and TRAIL is tightly controlled and restricted in physiological conditions to innate and adaptive immune system cells as well as to immune privilege sites, such as the eyes, the placenta, or the testis [12,13,14]. Both ligands are expressed at the surface of the two main immune effector cells, i.e., activated T cells and natural killer (NK) cells, but also on macrophages, neutrophils, and dendritic cells [15,16,17,18,19,20,21,22,23,24]. Their expression can be induced in response to TCR (T cell receptor) activation, but also upon cytokine stimulation particularly interferons (INF) through transcriptional regulation [16,18]. In contrast to their specific ligands, the TRAIL receptors and Fas are ubiquitously expressed inside and outside the immune system.

As TRAIL and FasL were initially described as apoptosis inducers, their death-inducing capacities and the associated molecular mechanisms were extensively studied [10,25]. Briefly, their binding to their respective cognate DD-containing receptors triggers the recruitment of several adaptor proteins that form a death-inducing signalling complex (DISC), which initiates the caspase activation and ultimately leads to the death of the sensitive target cell. This death-inducing function is mainly used by cytotoxic T cells and NK cells to eliminate the unwanted cells, such as cancer cells and virus-infected cells, but also autoreactive lymphocytes and activated lymphocytes during the contraction phase of an infection. In the latter, the involvement of the FasL/Fas system is particularly reported: the TCR restimulation through the upregulation of FasL expression leads to the death of Fas bearing clones in an antigen (ag) specific manner, a mechanism that is known as restimulation-induced cell death (RICD) [26].

It is now well established that, besides these well-known killing functions, FasL and TRAIL also generate other signalling pathways, leading to non-death functions or cell survival. In immune cells, they were reported affecting differentiation, migration processes, as well as cytokines production [27,28,29,30,31]. The molecular mechanisms underlying these non-death signals, although less well described than the apoptotic ones, start to be molecularly deciphered. Their description is beyond the scoop of this review, but it can be found in recent literature [32,33,34,35].

## 2. TRAIL and FasL Functions in the Control of Autoimmunity

The TRAIL and TRAIL-R knock out mice are viable and fertile [36,37]. In contrast to the FasL/Fas deficient mice, they do not spontaneously develop autoimmune diseases, but they rather present an increased severity of experimentally-induced autoimmune diseases (see Table 1). Autoimmune diseases can be induced by immunizing mice with specific ags, as it is the case for experimental autoimmune encephalomyelitis (EAE) (a mouse model of human multiple sclerosis), experimental induced thyroiditis (EAT), and collagen-induced rheumatoid arthritis (RA) or by the chemical treatment of mice in streptozotocin-induced type I diabetes. In these models, the activation of autoreactive T cells, mainly from CD4+ Th1 and Th17 subtypes, are the principal actors of the immune-mediated defects. An increase of the symptoms observed when inducing EAE [38,39,40], RA [41], or type I diabetes [41] in TRAIL or TRAIL-R deficient mice (demonstrated mainly by using the C57BL/6 TRAIL^−^/^−^ mice, C57BL/6 TRAIL-R^−^/^−^, but also the BALB/c TRAIL^−^/^−^ mice) when compared to their wild type counterpart clearly demonstrated the protective role of the TRAIL/TRAIL-R systems (Table 1). Moreover, intraperitoneal TRAIL injection decreases inflammation and autoimmune damages in mice, in which EAE or EAT was previously induced [38,42,43]. Conversely, TRAIL neutralization by the injection of either antibodies or soluble TRAIL receptor was shown to enhance the development of EAE [38,44], collagen-induced RA [45], as well as insulitis in non-obese diabetic (NOD) mice [46]. All together, these results identified TRAIL as a guardian against autoimmunity in several autoimmune diseases models.

**Figure 1:**
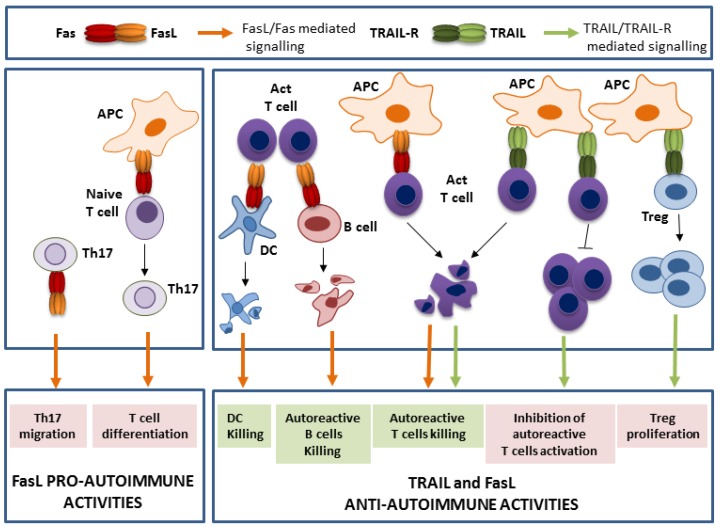
TRAIL and FasL activities in the control of autoimmunity. Whereas, TRAIL acts as a guardian against autoimmunity, FasL exerts both a role of guardian, but also promoter of autoimmunity. The main respective roles of their death (highlighted in green) and non-death (highlighted in pink) functions are represented. APC is for antigen presenting cells, DC for dendritic cells, and Act T cell for activated T cells.

The mechanism underlying these effects of TRAIL relies on its critical role in the maintenance of peripheral tolerance. Peripheral tolerance, a mechanism that is controlled by dendritic cells (DC), deals with the control of autoreactive immune cells at the periphery, in opposition to central tolerance, which consists in the deletion of developing autoreactive T cells or B cells in the thymus and bone marrow, respectively. Actually, Hirata et al. could prevent the development of EAE in mice by the transfer of DC presenting the auto ag and genetically modified to express TRAIL [47,48]. The same strategy was used to prevent the development of collagen II-induced arthritis [52]. TRAIL was described to control peripheral tolerance through the regulation of T cell compartment homeostasis by at least three different actions, leading to the neutralization of CD4+ autoreactive T cells (Figure 1). The main explanation of the protective role of TRAIL was initially attributed to an increased death of autoreactive T cells [47,52]. Later, it was also reported that TRAIL is able to stimulate the immunosuppressive regulatory T cells (Treg) proliferation by a so-far unknown mechanism, a process that leads to the inhibition of autoreactive T cell proliferation [43,48]. Indeed, Hirata et al. elegantly demonstrated that the adoptive transfer of Treg cells from mice transferred with TRAIL-expressing DC is sufficient to protect recipient mice from EAE induction [39,48]. Lastly, TRAIL was reported to directly inhibit the activation and proliferation of pathogenic Th1 or Th17 cells [38,39,40,47,58], notably by interfering with TCR proximal signal activation [40,58]. Nevertheless, the generation of cell specific TRAIL knockout mice would allow strictly identifying the precise role of each immune cell type in the anti-autoimmune function of TRAIL. However, it is important to remember that neither mutations nor modulation of the TRAIL/TRAIL-R expression have been reported so far in the etiology of autoimmune disease in humans.

In contrast, a human genetic disease, called autoimmune lymphoproliferative syndrome (ALPS), arises from mutation in the fas gene, in the fasl gene, or in genes encoding for effector proteins that are involved in Fas-induced cell death signalling, such as FADD (Fas-Associated protein with Death Domain) and caspases-8 or-10 [59]. ALPS patients present an uncontrolled lymphocyte proliferation that is accompanied by autoimmune manifestations, such as cytopenia or organ-specific autoimmunity, including hepatitis, glomerulonephritis, or dermatitis [60]. Notably, the naturally occurring lpr or gld mice, which bear mutations in the fas and fasl-encoding gene, respectively, are phenotypically similar to ALPS patients and they represent a model for autoimmune disease [61,62]. These mice develop progressive lymphadenopathy, splenomegaly, and an excess of immunoglobulins, including auto antibodies and systemic lupus erythematous (SLE) -like autoimmune symptoms. However, the severity of the phenotype depends on the genetic background [63]. The lymphocytes that accumulate in the secondary lymphoid organs predominantly present the pathognomonic TCRα/β CD4^−^ CD8^−^ double negative phenotype, even though the conventional CD4^+^, CD8^+^ T lymphocytes and B lymphocytes also show a several fold increase [63]. Altogether, these observations demonstrate a critical role of the FasL/Fas system in the prevention of autoimmunity and the control of lymphocytes homeostasis. Interestingly, TRAIL deficiency in a gld background significantly exacerbates both lymphoproliferation and autoimmune disease when compared to the gld mice, suggesting some redundancy between FasL and TRAIL and a role for these two receptors in the control of T cell homeostasis and autoimmunity [64].

The attribution of autoimmunity regulation to one specific cell type has been a debate for several years. However, it is now recognized that Fas expression on T cells, B cells, and DC is determinant for induction of peripheral tolerance. The first experiments conducted in lpr mice showed that the transgenic expression of Fas in their T cells is sufficient to prevent the lymphocytes accumulation (including the double negative T cells), but not the SLE development [65]. In contrast, the re-introduction of Fas in the lpr mice B cells avoids the SLE autoimmune manifestation, but do not prevent the T cell lymphoproliferation [66]. Additional critical informations came from tissue-specific deletion of Fas. Indeed, Stranges et al. reported that: (i) loss of Fas expression in T cells, which is critical for autoreactive T cell death recognizing low affinity self-ags, lead to autoimmune symptoms development and (ii) the conditional deletion of Fas in DC is sufficient to cause systemic autoimmunity, which is mainly due to DC accumulation and increased ag presentation [67] (Figure 1). They revealed a negative regulatory loop, in which T cells that recognize ags on the DC eliminate them by Fas-induced cell death to limit the immune response. On its side, the specific deletion of Fas in B cells lead to autoimmune disease, since Fas expression on B cells is involved in the negative selection of autoreactive B cells [67,68,69] (Figure 1). Interestingly, as B cells have also a role in ag presentation and thus T cell activation, the increased B cell number that is obtained due to lack of Fas-induced apoptosis in turn activates a large number of T cells, resulting in both B and T lymphoproliferation and exacerbating autoimmunity through the survival of autoimmune T cells [67,69].

More recently, some authors proposed that the control of the T cell compartment by Fas could be independent of its death function [70,71,72,73]. Fas palmitoylation is required for Fas localization in lipid raft, receptor aggregation and stability, and finally efficient cell death induction [74,75,76]. Palmitoylation-deficient mutant is defective in inducing death in primary mouse T cells, B cells, and DC, while retaining the capacity to trigger naïve T cell differentiation [72]. Interestingly, the palmitoylation-deficient receptor reintroduced in lpr mice reverses the lymphoproliferation and autoimmunity, suggesting that Fas does not protect from autoimmunity through its death-inducing capacity, but rather through its survival functions [72]. Additionally, Daszkiewicz et al. have suggested that Fas controls T cell homeostasis by inhibiting their proliferation following their observation of a reduction of effector and memory T cell accumulation and autoimmune associated symptoms upon the expression of p21 in T cells of lpr mice [73]. Indeed, FasL has been shown to induce p21 expression, and thus block the cell cycle progression of activated T cell, leading to the inhibition of their proliferation [71].

Paradoxically, while Fas has a suppressive effect on spontaneous systemic immunity, as seen with the development of autoimmunity in lpr or gld mice, Fas also plays a promoting role in the development of experimentally-induced autoimmune disease, since lpr or gld mice are resistant to EAE, RA, and type I diabetes induction [49,50,53,54,55,57] (Table 1). The promotion of EAE development by Fas was elegantly recently solved by Meyer Zu Horste et al., who revealed that Fas, through a death independent function, drives Th17 differentiation, which are the critical promoters of autoimmune tissue inflammation [51] (Figure 1). By transcriptional profiling analysis, Fas was shown to control the Th1/Th17 balance by a STAT-1 mediated mechanism [51,77]. Indeed, as both RA and type I diabetes development also involve Th17-mediated inflammation, the same molecular mechanism could be transposed and explain the role of Fas in supporting their progression. An additional autoimmune-promoting role of Fas involving Th17 has been recently described in SLE patients. The authors demonstrated that cleaved FasL in inflamed tissue chemoattracts Fas expressing Th17 cells and favors their transmigration across the endothelial barrier [78]. Despite intense research, controversies regarding the precise cellular etiology of the milder development of insulitis in NOD/lpr mice remain. Some of the authors suggest that the decreased β cells destruction that was observed was due to their own inability to signal through Fas upon binding to FasL from effector T cells [54]. However, others demonstrated that Fas deficiency on the T cells impaired their reactivity and thus initiates the phenotype [56].

In contrast to the role of TRAIL, which, to our current knowledge, seems limited in preventing autoimmunity through its action on different T cells subtypes, the contribution of the FasL/Fas system in autoimmune disease development reaches a high level of complexity, depending on the multiplicity of the cellular subsets involved and the multiplicity of functions activated by Fas triggering (Figure 1). While it can prevent autoimmune development by targeting T cells, B cells and DC, it can also promote it, notably through the differentiation of the pathogenic Th17 cells. The integration of all these informations is necessary to complete the general picture, which is far from being solved.

## 3. TRAIL and FasL Functions in Cancer Immunoediting

The TRAIL/TRAIL-R and FasL/Fas systems are critical players in the complex and reciprocal relationship that exists between cancer cells and the immune system, which is recapitulated within the cancer immunoediting concept. This dynamic process integrates the coexistence of promoting and constraining tumoral functions that are operated by different immune cells subtypes within the tumor environment through three consecutive key phases: elimination, equilibrium, and escape. The elimination phase relies on the removal of the malignant cells by both innate and adaptive immune cells, notably through perforin/granzyme and FasL/TRAIL-mediated killing. An immune selective pressure is maintained on the tumor cells that had survived this first surveillance stage, during which the highly resistant clones are selected. Therefore, they could escape from the immune system thanks to their abilities to survive in an immunosuppressive environment, proliferate, and disseminate in other organs, which can be mediated in part by TRAIL and FasL-induced signalling. The demonstration of the critical implication of the immune system in the tumor development has allowed great advances in the design of therapies, namely immunotherapies, in which TRAIL and FasL signalling could be targeted.

### 3.1. Role of TRAIL and FasL in the Elimination Phase

The evidence for the involvement of the endogenous TRAIL/TRAIL-R system in tumor elimination came from animal studies using TRAIL^−^/^−^ [36,79,80] or TRAIL-R^−^/^−^ mice [81,82,83] or mice that were injected with TRAIL neutralizing antibodies [84,85,86] and mostly conclude to a protective role of the TRAIL/TRAIL-R system against cancer development (Table 2).

In TRAIL^−^/^−^ mice, an increased susceptibility to both tumor initiation and metastasis development was demonstrated by several experimental methods, including transplantation of syngenic cancer cell lines, the spontaneous occurrence of late age lymphoma or the development of chemical carcinogen methylcholanthrene-induced fibrosarcomas [36,79,80,84]. The TRAIL-R invalidation leads to more contrasting results: on one hand, the TRAIL-R deficiency was found to recapitulate the TRAIL deficient phenotype, since the loss of TRAIL-R in the lymphoma-prone Eμmyc genetic background significantly reduced the lymphoma-free survival and enhanced diethylnotrosamine (DEN)-induced hepatocarcinogenesis [81]. On the other hand, Yue et al. identified no tumor promotion in TRAIL-R^−^/^−^ mice, even when the deficient mice were crossed with p53^−^/^−^ mice or APC ^min^/^+^ mice [82]. The discrepancies that were observed between this work and others backcrossing with p53^+^/^−^ mice could be due to the complete loss of p53, which might affect the TRAIL/TRAIL-R system expression and function. A study performed by Grosse-Wilde et al. brings interesting clues: the authors reported that, whereas an increased lymph node metastasis is detected in TRAIL-R deficient mice that were subjected to the DMBA/TPA-induced model of squamous cell carcinomas, no incidence is found in the primary tumor. Interestingly, while the adherent primary tumor cells were resistant to TRAIL-mediated cell death, their detachment, which is a mandatory step in metastasis formation, sensitize them to TRAIL-induced apoptosis and provide an explanation for the role of TRAIL as a metastasis suppressor [83]. This finding can be read in light with a recent work from our laboratory demonstrating that inhibition of cell-cell adhesion of epithelial cells sensitizes them to Fas-induced cell death [91]. A sequestration of Fas by cadherins in adherens junctions, as well as the association of Fas and the polarity molecule Dlg1, which inhibits DISC formation, molecularly account for the inhibition of Fas-induced cell death in polarized cells. Thus, it is tempting to speculate that similar molecular control of TRAIL-R localization and association could account for their sensitization to TRAIL upon the loss of adherence.

The role of the FasL/Fas system in preventing cancer development was highlighted in ALPS patients who present a higher incidence of non-Hodgkin and Hodgkin lymphoma [92]. It is more difficult to assess in mice models due to the altered immune system that results from FasL/Fas deficient signalling. Nevertheless, some studies demonstrated that aging gld mice develop spontaneously malignant plasmacystoid lymphomas, even though the late development of the tumors strongly suggests that additional genetic mutations are necessary for malignant transformation to occur [89] (Table 2). Actually, no spontaneous occurrence of cancer development was reported in lpr mice, but the loss of Fas was shown to accelerate the lymphoma development in E mu L-myc transgenic mice [88]. In addition, T cell deficient lpr mice develop intraperitoneal B cell lymphoma and the authors propose that the loss of Fas promotes a pool of premalignant B cells that progress to malignancy in the absence of T cells [87].

**Figure 2:**
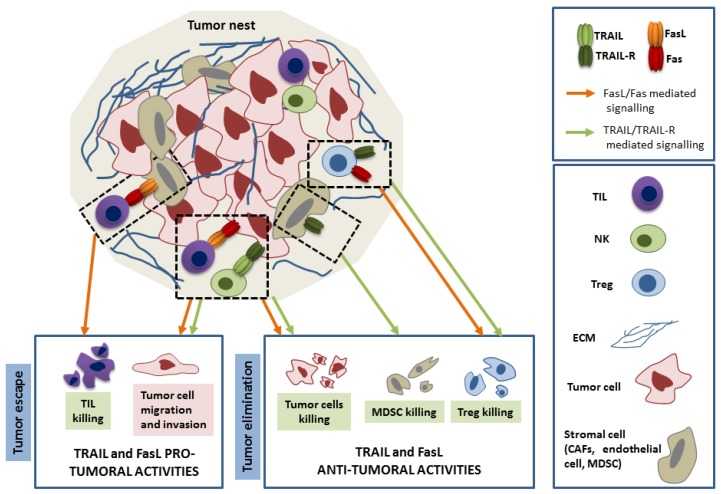
TRAIL and FasL pro- and anti-tumoral activities within the tumor nest. The main respective roles of their death (highlighted in green) and non-death (highlighted in pink) functions are represented. Briefly, TRAIL and FasL, mainly from tumor-infiltrating lymphocytes (TIL) and natural killer (NK) cells origin target the different cells of the tumor nest. They trigger the death of immunosuppressive cells such as regulatory T cells (Treg) and myeloid-derived suppressor cells (MDSC). The tumor cells can respond either by death but also non death pro tumoral functions which lead to tumor escape. Moreover, the tumor can escape from the immune system by specific killing of the TIL through abnormal expression of FasL on stromal cells.

Cytotoxic T lymphocytes (CTL) and NK cells are the two mains players in the immune-mediated elimination of cancer cells. Even though they are activated by different ways, they use the same mechanisms to kill their target: (i) the granule exocytosis pathway using the pore-forming protein perforin and the serine proteases granzymes and (ii) the expression of the death ligands FasL and TRAIL. The origin of the protective role of TRAIL in cancer development has been mainly attributed to its cytotoxic function on hepatic NK cells (Figure 2). Indeed, while neutralizing TRAIL with anti-TRAIL antibodies significantly enhance metastasis formation in mice, previous NK cells depletion abolished this effect, indicating that the cytotoxic activity of TRAIL expressed on NK cells is responsible for its anti-metastatic action [85]. Moreover, the ex vivo mediated NK cells cytotoxicity was dramatically reduced in TRAIL^−^/^−^ mice when compared to their wild type counterparts, leading to the conclusion that TRAIL, as well as FasL and perforin, contribute to the anti-metastatic effect of the NK cells [80]. Importantly, TRAIL contributes to the INFγ-mediated anti-metastatic effect of NK cells, probably through the upregulation of TRAIL expression upon INFγ treatment [86]. Nevertheless, the contribution of other cell types to TRAIL-mediated anti-cancer effect has been demonstrated and TRAIL has been reported as a mediator of tumor-specific CD4+ cytotoxic lymphocytes-mediated cell death in lung cancer [93]. Importantly, not only the cancer cell killing, but also the targeting of tumor supportive cells that are present within the tumor nest, such as myeloid-derived suppressor cells (MDSC) and Treg, might account for some anti-cancer role of TRAIL [94,95] (Figure 2).

If the perforin-granzyme system has been shown to play a major role in tumor cell elimination, as demonstrated by the development of spontaneous B cell lymphomas in the perforin-deficient mice, FasL expressed on CTL is a second way used by lymphocytes to kill cancer cells (Figure 2). Indeed, while in perf^−^/^−^ mice an anti-tumor response is still carried out by the CTL, this cytotoxic effect cannot be seen in ex vivo experiments where FasL has been neutralized [96]. Therefore, both systems appear to be critical and complementary to optimally achieve tumor rejection [96,97,98]. However, the exact contribution of these two cytotoxic pathways used by CTL to kill the cancer cells is still unsolved. Shanker et al. propose that the ag concentration presented by tumor cells might determinate the type of system used: the FasL system might be prominently used when a limited amount of ag is presented by the tumor cells, while the perforin system might become more present when this amount increases [99]. This hypothesis is coherent with previous studies that were done in a cancer-independent context, describing that a weak TCR signalling is able to activate a FasL, but not a perforin-mediated CTL death [100]. Interestingly, a recent study questioned the role of the immune system in controlling the development of B lymphoma. Afshar-Sterle et al. not only show that the lymphoma development was inhibited by different CD8+T cell clones presenting a large TCR range, but also identified FasL expression on their surface as a way of limiting the lymphoma development [90]. Based on these observations, the authors develop an interesting hypothesis that could explain the unique lymphoma nature of cancers that developed in a deficient FasL/Fas context: since B lymphoma cells could behave as APC, T cells bearing a low affinity TCR for self-ags eliminate through a FasL/Fas system the developing B lymphoma cells, an elimination that cannot be taken over in lpr and gld mice or in ALPS patients.

### 3.2. Role of TRAIL and FasL in Immune Escape

The cancer cells use different strategies to escape from the NK cells and CTL-mediated killing. Amongst them, the apoptosis of tumor-infiltrating lymphocytes (TILs) represents an effective way to prevent anti-tumor immunity and it can be reached by the death ligand expression within the tumor microenvironment and the death receptor expression on the TIL. This old idea of tumor counterattack gave rise to intense debates that were initiated 20 years ago. Some clarifications recently came from several studies that showed that an abnormal FasL expression within the tumor is playing an active role in the tumor development by allowing the tumor to escape. They identified the cellular origins of the FasL expression within the tumor microenvironment: in contrast to what was initially thought, FasL was not expressed at the surface of the tumor cells, but by endothelium cells, cancer-associated fibroblast (CAFs), and myeloid-derived suppressor cells (MDSC) [101,102,103]. Indeed, FasL neutralization in each of these three cell populations lead to the Fas expressing TILs inhibition of apoptosis and increased tumor rejection. Such a clear demonstration has not been reported so far for TRAIL. Nevertheless, the observation relating a correlation between the rate of apoptotic TILs and their TRAIL-R1 expression suggest that TRAIL-R1-mediated TIL apoptosis could also participate to tumor immune escape, a hypothesis that is coherent with the expression of TRAIL by the colorectal tumor cells [104].

The tumor cells themselves could adapt to counteract the death signal that is induced by TRAIL-R or Fas activation by down regulating their surface expression. A decreased expression of TRAIL-R1 upon TGFβ (Transforming growth factor beta) treatment has been detected in pancreatic cancer cell lines and correlates with a decrease in TRAIL-induced cell death [105]. Afshar-Sterle et al. demonstrate that B-lymphoma which develops in a T cell sufficient mouse model has a weaker Fas expression when compared to those arisen from T cell deficient mice and are more resistant to FasL-induced apoptosis [90]. In the case of TRAIL, the overexpression of the decoy receptors TRAIL-R3 and TRAIL-R4 have been reported in some tumor types (including acute myeloid leukemia), where they might sequester TRAIL, but also form heteromers with TRAIL-R1 and TRAIL-R2, thereby preventing TRAIL-R1 and TRAIL-R2 activation and proper DISC formation [25,106]. The overexpression of DcR3 has also been reported in tumors, such as colon and lung [25]. However, the consequence on Fas signalling is more difficult to assess, since it also acts as decoy receptor for TL1A and LIGHT and presents independent non-decoy functions. Additionally, some tumor cells also modulate their expression of essential mediators of cell death signalling, as it is the case for the anti-apoptotic regulator c-FLIP, which is found to be frequently overexpressed in tumors of different origins [107].

Cancer cells not only develop resistance to FasL and TRAIL-mediated immune cell death, but they also highjack the TRAIL and FasL non–death signalling pathway in order to support migration and invasion. This is mainly true in the presence of an oncogenic mutation. Both FasL and TRAIL were reported to stimulate the invasion of colorectal tumor cells and liver metastasis in mice through a K-Ras -dependent way [108]. The authors demonstrated that mutated K-Ras and its effector Raf-1 can switch the death receptor signalling from death to invasion through the inhibition of the Rho/ROCK/LIMK pathway. More recently, Von Karstedt et al. demonstrated, via the cancer cell restricted genetic ablation of TRAIL-R, that spontaneous metastasis in a K-Ras driven pancreatic ductal adenocarcinoma requires the expression of TRAIL-R [109]. Working in human cell lines, they only identified TRAIL-R2, but not TRAIL-R1, as the driver of this cancer progression, but also reported the critical importance of the membrane proximal domain of TRAIL-R2 to induce a Rac1 mediated pro-migratory signalling pathway. Interestingly, a kinome analysis of the non-death pathway induced by TRAIL in non-small-cell lung carcinoma identified a RIPK1/Src/STAT3 pathway that was only activated by the TRAIL-R2 receptor and lead to cell migration and invasion [110]. Thus, the two TRAIL receptors might have different abilities in activating cancer-promoting functions. The recruitment of the Src kinase Yes and the p85 PI3K subunit to Fas upon FasL activation was demonstrated in glioblastoma cells and it could give rise to an invasion signal through the activation of the Akt/GSK3β axis, which leads to subsequent matrix metalloproteases expression [111]. Neutralizing the FasL pathway in mice dramatically reduces the number of invading cells [111]. A recent report from our laboratory demonstrated that Src kinase-mediated phosphorylation of Fas within its death domain is a key event in switching Fas signalling from death to non-death: the dephosphorylation of both tyrosines in the death domain of Fas by the SHP-1 phosphatase turns on the apoptotic signal, whereas the tyrosine phosphorylation turns off the pro-apoptotic signal and turns on the prosurvival. Furthermore, we provide evidence that Fas tyrosine phosphorylation status may vary among different cancer types and influence the response to anti-cancer treatments [112].

## 4. TRAIL and FasL in Clinical Interventions

Huge expectations in the use of TRAIL in cancer treatment were generated, since systemic administration of TRAIL in mice was shown to be not only effective in killing human breast or colon xenografted tumor cells, but also in causing less toxicity than FasL or TNF administration [113]. Based on these initial observations, TRAIL was considered as the most promising tumor selective ligand, and intense world-wide research efforts were made on TRAIL-R agonists as potential novel cancer therapeutics.

Nevertheless, this initial optimism to target TRAIL for anticancer therapy was stunted by the disappointing results that were obtained with the TRAIL-R agonists clinical trials that, although confirming the good tolerance of the treatment, did not show any robust benefits for patients [106,114]. Two classes of molecules have been tested so far in clinic, recombinant form of the ligand (i.e., Dulanermin) or agonistic antibodies targeting the receptors (i.e., TAS266, AMG-655) [106]. The lack of success for TRAIL targeting molecules was mainly attributed to an insufficient agonistic activity, a short half-life of the molecule in situ, and the resistance of the majority of cancer cells to TRAIL-induced cell death. Several attempts have been made to overcome these difficulties. Recently, a combination of recombinant TRAIL and agonistic TRAIL-R2 was shown to synergize in the killing of cancer cells via enhanced multimerization of TRAIL-R2, which brings new hope for their use in therapy [115]. New TRAIL formulations with increased activity have been designed and evaluated in preclinical studies [116]. They aim at increasing the stability of the molecules, but also the specific targeting to cancer cells [116]. If TRAIL system targeting drugs were not yet tested in auto-immune disease contexts, the progress that has been made in cancer therapy could benefit to auto-immune affected patients and the activation of the TRAIL pathway could be validated as a therapeutic option.

The idea to use the FasL system as a potential therapeutic intervention was abandoned when it was described that activating Fas antibody injection in mice induces hepatocyte apoptosis, liver failure, and death. It is now known that the main cause of such toxicity is attributed to the Fc part of the antibody [117,118]. Later, recombinant FasL or Fas molecules have been designed. One of them, called megaFasL or APO010, is a synthetic hexameric Fas agonist that is generated by the fusion of two trimeric FasL, is currently in clinical trials evaluation after successful assays in animal models [119]. A second molecule, APG101, which consists of the extracellular domain of Fas fused to the Fc domain of IgG binds FasL, thereby acting as an antagonist, is in a phase II clinical trial for glioblastoma treatment [120,121].

Importantly, some critical questions regarding the death receptor targeting in cancer treatment remain. First, as TRAIL and FasL are both involved in the death of primary cells, such as hepatocytes, immune cells, or neurons, the administration mode and the molecule design has to be carefully thought. Secondly, the question of blocking or activating Fas or TRAIL-R signalling is still opened. The TRAIL-R agonists, by inducing apoptosis of cancer cells, regulatory T cells, and MDSC, could represent a real improvement for cancer therapy. In the other hand, in some other tumors, such as those that bear the mutated K-Ras, neutralizing the TRAIL/TRAIL-R axis might exert a beneficial effect on tumor development. The same question arises for Fas. On the contrary to what has been initially anticipated, several independent studies came to the conclusion that FasL/Fas axis neutralization would extend the benefits of cancer immunotherapy: (i) one major role of FasL/Fas in the immune cells is to promote the RICD of activated T lymphocyte at the end of the immune response. Blocking this function would improve T cell persistence at the tumor site; (ii) the expression of FasL by some stromal cells has been shown to trigger TILs apoptosis therefore participating to the immunotherapy resistance [101,102,103]; (iii) Fas expressed on memory T cells induces the precocious differentiation of naïve Fas-expressing T cells that limits their anti-tumor action through an Akt-dependent pathway [122]. Thus, neutralizing the FasL/Fas axis could synergize with several immunotherapy strategies by improving T cell activity. However, maintaining or even enhancing the FasL/Fas activity might also be of high interest by potentiating the T cell-mediated cytotoxicity, an important process that achieves tumor rejection. In conclusion, major precautions need to be taken before applying a FasL/Fas blockage strategy that could definitively alter the efficient T cell activation, and thus the anti-cancer response.

Important lessons that arise from the disappointing translation of death ligands mouse model studies into human therapeutic must be learnt in order to improve the design of future drugs. First of all, mouse and human death receptor/ligand systems display major differences in terms of sequence, number and nature of receptors, and post-translational modifications, which make the comparison of the systems and adaptation of results obtained from mouse in humans difficult. Moreover, despite the proper expression of TRAIL-R or Fas on tumor cells, the response to the receptors activation may vary extremely from death-induction, no response, to tumor promotion. Consequently, the identification of valid biomarkers that are able to predict the response of each patient to TRAIL-Rs or Fas stimulation is crucial to determine which patient will benefit from such treatment. In conclusion, a better understanding of the molecular mechanisms underlying TRAIL or FasL-mediated signalling pathways in human is necessary to bring death-receptor-based therapy to success.

## 5. Conclusions

As described in this review, whereas TRAIL is only known for its anti-autoimmune activities through targeting several T cell subsets, the situation is more complicated for FasL, which presents both pro and anti-autoimmune functions. In the context of cancer, both FasL and TRAIL are involved in killing cancer cells, but whereas the role of FasL mainly seems attributed to its expression on cytotoxic T cells, the role of TRAIL seems to rely on its expression on NK cells. In this way, FasL and TRAIL may exert complementary functions that involve both adaptive and innate immune cells in order to more efficiently limit the cancer cells expansion. On the other hand, the role of both molecules in tumor escape is now well documented, notably by their abilities to induce tumor cell migration.

In conclusion, despite a similar cellular expression pattern and comparable molecular signalling mechanisms used by their receptors, the individual participation of TRAIL and FasL in autoimmunity and cancer development are mainly not redundant, but rather complementary. A deep analysis of their precise role revealed a huge complexity that arises from the different cell subsets that are involved either as effector or targets. A second level of complexity is added by the different outcomes that can be triggered by TRAIL and FasL signalling, since, in the two pathological contexts, both the death and the non-death activities of the ligands are involved. This general complex picture is important to keep in mind, especially when considering the targeting of the TRAIL/TRAIL-R and FasL/Fas systems in therapeutic strategies.

## Figures and Tables

**Table 1:** The role of Tumor Necrosis Factor-Related Apoptosis Inducing Ligand (TRAIL)/TRAIL-R and Fas Ligand (FasL)/Fas systems in experimentally-induced autoimmune disease mice models.

Autoimmune Mouse Model	Ligand /Receptor Status	Outcome	References
Experimental autoimmune encephalomyelitis (EAE)	TRAIL neutralization (sDR5)	Exacerbation of symptoms	[44]
	TRAIL neutralization (TRAIL Abs)		[38]
	TRAIL ^−^/^−^ mice		[38,39]
	TRAIL-R ^−^/^−^ mice		[40]
	TRAIL injection	Attenuation of symptoms	[38]
	TRAIL expressing DC		[47,48]
	lpr mice		[49,50,51]
	gld mice		[49,50]
Experimental autoimmune thyroiditis (EAT)	TRAIL injection	Attenuation of symptoms	[42,43]
Collagen-induced rheumatoid arthritis (RA)	TRAIL neutralization (sDR5)	Exacerbation of symptoms	[45]
	TRAIL ^−^/^−^ mice		[41]
	TRAIL expressing DC	Attenuation of symptoms	[52]
	lpr mice		[53]
Type 1 diabetes	TRAIL neutralization (sDR5)	Exacerbation of symptoms	[46]
	TRAIL ^−^/^−^ mice		[41]
	NOD lpr mice	Attenuation of symptoms	[54,55,56]
	NOD gld mice		[57]

**Table 2:** The role of TRAIL/TRAIL-R and FasL/Fas systems in cancer development in mice models.

Ligand/Receptor Status	Cancer Induction	Outcome	References
TRAIL ^−^/^−^ mice	Spontaneous	Late-age lymphoma	[79]
	p53 ^+^/^−^ mice	Sarcoma, lymphoma	[79]
	Her2/neu mice	No symptoms	[79]
	A20 cell line transfer	Lymphoma	[36]
	Renca cell line transfer	Liver metastasis	[80]
	4T1 cell line transfer	Mammary carcinoma Lung and liver metastasis	[80]
	MCA induction	Fibrosarcoma	[80]
TRAIL neutralization by Abs	p53 ^+^/^−^ mice	Sarcoma, lymphoma	[84]
	L929 cell line transfer	Liver metastasis	[85]
	Renca cell line transfer	Liver metastasis	[86]
	MCA induction	Fibrosarcoma	[84]
TRAIL-R ^−^/^−^ mice	Eu-myc mice	Lymphoma	[81]
	p53 ^−^/^−^ mice	No symptoms	[82]
	APC ^min^/^+^ mice	No symptoms	[82]
	DMBA/TPA	Lymph node metastasis	[83]
	DEN treatment	Hepato carcinoma	[81]
lpr mice	T cell deficient	Lymphoma	[87]
	Eu-myc mice	Lymphoma	[88]
gld mice	Spontaneous	Lymphoma	[89]
FasL^−^/^−^ T cells (CD8+)	Lymphoma cells transfer in rag1^−^/^−^ mice	Lymphoma	[90]
